# NOVEL REMOTE ASSESSMENT OF THE STANDING POSTURAL CONTROL IN YOUNGER AND OLDER ADULTS USING SMARTPHONE APPLICATION

**DOI:** 10.1093/geroni/igz038.1216

**Published:** 2019-11-08

**Authors:** Junhong Zhou, Wanting Yu, Hao Zhu, On-Yee Lo, Thomas Travison, Lewis Lipsitz, Brad Manor

**Affiliations:** 1 Hinda and Arthur Marcus Institute for Aging Research, Harvard Medical School, Roslindale, Massachusetts, United States; 2 Hebrew SeniorLife, Roslindale, Massachusetts, United States

## Abstract

In older adults, assessment of standing postural control under various task and/or environmental conditions provides valuable insight into cognitive-motor function. To date, however, such assessments have been limited primarily to laboratory or clinical settings. We therefore created a smartphone App to enable remote assessments of postural control. This App provides users with standardized multi-media instructions and harnesses the phone’s internal motion sensors to capture postural sway, with the phone placed in the user’s pants pocket, during trials of standing with eyes open, eyes-closed, and while performing serial-subtractions (i.e., dual tasking). We then established the test-retest reliability of several metrics of postural sway derived from this assessment tool, as well as their sensitivity to the effect of age and standing condition. Fifteen healthy younger and 15 older adults completed multiple standing trials in two separate laboratory visits and on three separate days in their own homes. Postural sway metrics included the mean distance from the center of the trajectory and root mean square were derived from both transverse-plane acceleration and angular velocity time series. Each sway metric demonstrated excellent test-retest reliability, even when analyzed separately by group and standing condition (ICCs: 0.78-0.89). Moreover, each metric was sensitive to age group and standing condition, such that greater sway was observed in older adults as compared to younger adults (p<0.03), and in more challenging standing conditions (p<0.0001). These results suggest that sensitive metrics of standing postural control may be reliably obtained from remote smartphone-based assessments in both younger and older adults.

